# Transcriptome-based analysis of oil accumulation pattern and key gene screening in *Gardenia jasminoides* fruits

**DOI:** 10.3389/fpls.2026.1774066

**Published:** 2026-03-06

**Authors:** Liu Su, Jiwu Cao, Yunzhu Chen, Peiwang Li, Jingzhen Chen, Changzhu Li, Qiang Liu, Zhihong Xiao, Huifang Cao, Ding Kuang, Aihong Wu, Yudong Lu, Xiao Zhou, Yan Yang

**Affiliations:** 1 State Key Laboratory of Utilization of Woody Oil Resource, Hunan Academy of Forestry, Changsha, China; 2 College of Forestry, Central South University of Forestry and Technology, Changsha, China; 3 Key Laboratory of National Forestry and Grassland Administration on Utilization Science for Southern Woody Oilseed, Hunan Academy of Forestry, Changsha, China; 4 College of Environmental Engineering, Changsha Environmental Protection Vocational College, Changsha, China; 5 Hunan Haitai Bonong Biotechnology Co., Ltd., Yueyang, China; 6 Real Estate Registration Center, Xinning County Natural Resources Bureau, Xingning, China; 7 College of Chemistry and Materials Science, Fujian Normal University, Fujian, China

**Keywords:** fatty acid, *Gardenia jasminoides*, key enzyme genes, oil biosynthesis, transcriptome analysis, triacylglycerol

## Abstract

*Gardenia jasminoides* fruit is a highly promising woody oil resource, characterized by high oil content and a lipid profile enriched in unsaturated fatty acids with pharmacological activities such as cholesterol-lowering and antioxidant effects. To elucidate the molecular mechanisms underlying its oil accumulation, we systematically investigated fruit morphology, oil content, fatty acid composition, and oil body structure during development, and performed transcriptomic analyses at five key stages: 15, 45, 75, 105, and 150 DAF. These analyses revealed the developmental progression of the fruit, the patterns of oil accumulation, and the dynamic changes in fatty acid composition. DEGs analysis further elucidated the oil biosynthesis pathway and identified several key candidate genes. The results showed that *G. jasminoides* fruit development comprises three major stages: a rapid expansion stage (15~60 DAF), a color-transition stage (60~150 DAF), and a maturation stage (150~180 DAF). The fruit color gradually changed from green to orange-yellow and finally to orange-red, reaching morphological maturity at 150 DAF. Oil content exhibited an S-shaped growth pattern, reaching its maximum of 16.7% at 180 DAF. In mature fruits, the dominant fatty acids were linoleic acid (C18:2) and oleic acid (C18:1), with average relative contents of 55.2% and 20.6%, respectively, and unsaturated fatty acids accounting for 75.8% of main fatty acids. Oil body diameter displayed a distinct developmental pattern, increasing rapidly from 15 to 150 DAF, stabilizing between 150 and 180 DAF, and reaching a maximum of 29.8 μm at 180 DAF. Integrating fatty acid dynamics with DEG analysis, we screened *ACC*, *SAD*, *FATA*, *FAD2*, *DGAT2*, and *LACS2* as key candidate genes involved in oil biosynthesis in *G. jasminoides* fruit. Together, the transcriptomic analyses uncovered the molecular regulatory mechanisms of oil accumulation and enabled the construction of a metabolic pathway model for oil biosynthesis in *G. jasminoides*. These findings provide important theoretical insights and practical implications for enhancing oil yield and improving oil quality in *G. jasminoides*.

## 1 Introduction

The *Gardenia jasminoides*(*G. jasminoides*), also known as Gardenia, is an evergreen shrub belonging to the Gardenia genus in Rubiaceae (Chen et al., 2020). Gardenia, with its white and intensely fragrant blossoms, is widely used both as cut flowers for decoration and in landscape gardening (Çelikel et al., 2020). Its ripe fruit, which is orange-red in color, is commonly used in China for making tea, cooking congee, or naturally enhancing the color of dishes (Li et al., 2020; Wang et al., 2021). Furthermore, Gardenia holds significant medicinal value, as its fruit, leaves, flowers, and roots can all be used in herbal medicine (Zhang et al., 2022). The dried mature fruit, recognized as a dual-purpose resource for both food and medicinal applications, was included in the first list of medicinal and edible substances promulgated by the Ministry of Health of China (Chen et al., 2008).

Gardenia fruit is not only rich in oil, but also contains active ingredients such as geniposide and crocin (Debnath et al., 2011). There are many methods for extracting Gardenia oil, including Cold Pressing, Solvent Extraction, Supercritical Fluid Extraction, and Ultrasonic Assisted Extraction, each with its own advantages and limitations (Xiao et al., 2017). The oil content of Gardenia fruit is about 20%, which can be used for edible oil production (Chyau et al., 2022). Its most prominent nutritional and health value lies in its excellent composition of fatty acids, including linoleic acid (C18:2), oleic acid (C18:1), palmitic acid (C16:0), etc., with linoleic acid (37.0%~43.8%) accounting for the highest proportion (Jin et al., 2023). C18:1 is an essential fatty acid for the human body, which has functions such as regulating blood pressure and reducing serum cholesterol. In addition, numerous studies had isolated and identified various bioactive compounds from Gardenia, mainly including geniposide, geniposidic, genipin, crocins. These compounds have been reported to possess various physiological functions, including anti-inflammatory, antioxidant, antihypertensive, antihyperglycemic, anticancer, antihyperlipidemic, neuroprotective, and hepatoprotective effects (Yin et al., 2018). It also shows broad application prospects in regulated health food, high-end cosmetics, and biopharmaceuticals. However, the mechanism of oil synthesis in Gardenia fruits remains unclear. Therefore, elucidating the oil accumulation patterns and molecular regulatory mechanisms in Gardenia fruit would provide theoretical guidance for improving oil yield and optimizing fatty acid composition, as well as a scientific basis for the exploitation of high-value germplasm resources, molecular breeding, and cultivation practices.

Triglycerides (TAG) are the primary storage form of plant seed oils, and its biosynthesis and accumulation directly influence the economic value of oilseed crops (Zhou L et al., 2024). Oil synthesis involves three core stages: *de novo* fatty acid (FA) synthesis, acyl chain elongation, and TAG assembly,which are precisely regulated by multiple enzyme systems. The *de novo* synthesis in plants occurs in plastids. The process begins with the carboxylation of acetyl-CoA to malonyl-CoA, catalyzed by Acetyl-CoA carboxylase (ACCase). Subsequently, the FA synthase complex then uses malonyl-CoA as a two-carbon donor to elongate the fatty acid carbon chain through cycles of condensation, reduction, dehydration, and re-reduction. This ultimately produces predominantly palmitoyl-ACP (C16:0-ACP). Subsequently, the enzyme 3-oxoacyl-ACP synthase: KAS II (KAS II) acts as the key catalyst, converting C16:0-ACP into stearoyl-ACP (C18:0-ACP) (Bates et al., 2013). During the acyl chain modification stage, acyl-ACP desaturase (SAD) catalyzes medium- and long-chain saturated acyl-ACP substrates and introduces the first double bond at a specific position of the carbon chain, thereby producing unsaturated fatty acids such as C18:1-ACP.

Subsequently, fatty acyl-ACP thioesterase A (FATA) and fatty acyl-ACP thioesterase B (FATB) catalyze the hydrolysis of fatty acyl-ACP to release free fatty acids (FFAs). After synthesis, FFAs are transported from plastids to the endoplasmic reticulum for further involvement in lipid synthesis (Koo et al., 2004; Li-Beisson et al., 2013). During this export process, long-chain acyl-CoA synthetase (LACS) activates FFAs to form fatty acyl-CoA, which serves as a key substrate for the final assembly of TAG in the endoplasmic reticulum (Schnurr et al., 2002). The core pathway for TAG synthesis is the Kennedy pathway, which involves four sequential acylation steps: First, sn-glycerol-3-phosphate is acylated at the sn-1 position by glycerol-3-phosphate acyltransferase (GPAT) to produce lysophosphatidic acid. Next, Lysophosphatidic acid acyltransferase (LPAT) catalyzes the second acylation at the sn-2 position to form Phosphatidic acid (PA). Subsequently, PA is dephosphorylated by Phosphatidic Acid Phosphatase (PAP) to generate diacylglycerol. Finally, diacylglycerol O-acyltransferase (DGAT) catalyzes the third acylation at the sn-3 position, completing TAG synthesis (Gibellini et al., 2010). It is also noteworthy that ω-6 FA desaturase (FAD2) and ω-3 FA desaturase (FAD3) further introduce double bonds into free fatty acids or phospholipid substrates, regulating the ratio of polyunsaturated fatty acids, such as C18:2 and C18:3. This regulation significantly influences the unsaturation level and functional characteristics of oils. Additionally, the efficiency of TAG assembly primarily relies on the coordinated and synergistic action of DGAT and phospholipid: diacylglycerol acyltransferase (PDAT). DGAT uses ac tivated acyl-CoA to synthesize TAG, while PDAT uses phosphatidylcholine (PC) as an acyl donor for TAG synthesis (Lee et al., 2019).

Studies have revealed that during seed development, the genes encoding enzymes responsible for fatty acid synthesis and those involved in TAG assembly exhibit markedly distinct temporal expression patterns. However, the regulation of gene expression and enzyme activity associated with the late stages of oil biosynthesis remains poorly understood (Troncoso-Ponce et al., 2011; Bourgis et al., 2011). The molecular mechanisms underlying oil biosynthesis and accumulation in *G. jasminoides*, a promising medicinal and edible plant, remain poorly understood. To elucidate the unique oil and fatty acid composition characteristics of Gardenia fruit, this study used integrated transcriptomic analyses to reveal distinct expression patterns of genes involved in lipid biosynthesis.

Using Gardenia fruits from 15~180 days after flowering (DAF) as experimental materials, this study systematically observed the morphological characteristics during fruit development. And it also quantitatively analyzed the oil content and fatty acid composition, aiming to explore the patterns of oil accumulation and dynamic changes in fatty acids over time. Simultaneously, RNA-seq analysis was conducted at five key stages of Gardenia fruit development and oil accumulation to comprehensively elucidate the patterns of lipid accumulation and the molecular regulatory mechanisms underlying its biosynthesis. This study will provide potential candidate genes for the genetic improvement of *G. jasminoide*s using molecular biotechnology, offer theoretical guidance for its cultivation management, and lay a theoretical foundation for optimizing the oil yield and quality of its fruits.

## 2 Results

### 2.1 Developmental characteristics of gardenia fruits

**Figure 1:**
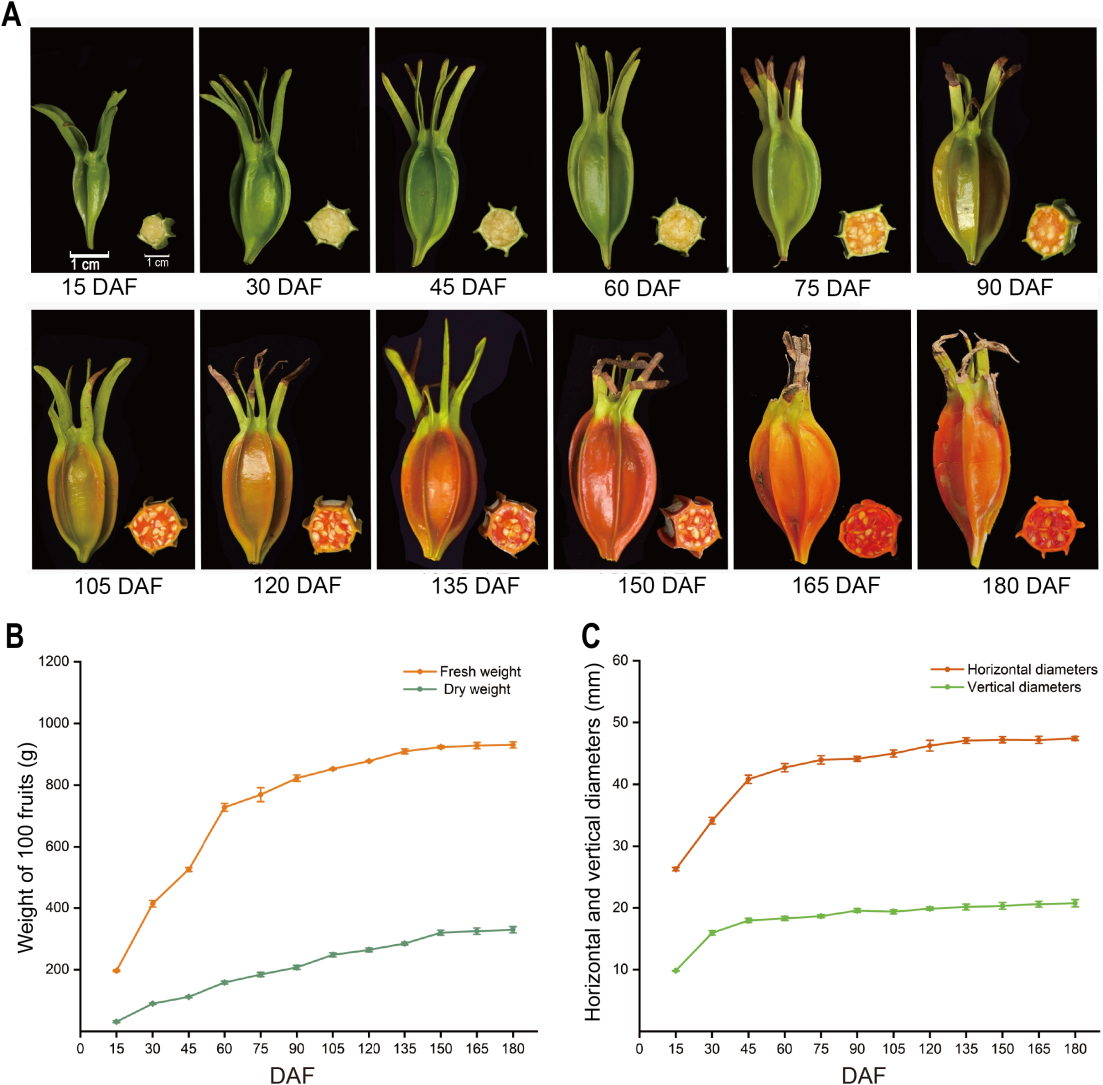
Dynamic change of *G. jasminoides* fruits at different developmental stages: **(A)** Morphological changes of *G. jasminoides* fruits during 12 developmental stages. **(B)** Changes in fresh and dry weight of *G. jasminoides* at different stages. **(C)** Changes in longitudinal and transverse diameters of Gardenia at different developmental stages.

The size and peel color of Gardenia fruits undergo significant changes during different stages of development. Fruit development can be divided into three stages: from 15 DAF to 60 DAF, which is the rapid enlargement stage, during which the peel changes from green to yellow-green, and the flesh becomes pale yellow. The fruit undergoes significant enlargement, with fresh weight and dry weight increasing rapidly. Fresh weight increases from 196.51 g to 727.77 g, and dry weight increases from 30.89 g to 158.45 g. The longitudinal diameter increases rapidly from 26.28 mm to 42.71 mm, and the transverse diameter increases from 9.83 mm to 18.29 mm. From 60 DAF to 150 DAF, the fruit enters the color change phase, where growth slows down. The fruit peel changes from green to orange-yellow, and the flesh changes from light orange to orange-red. Fresh weight increases from 727.77 g to 923.38 g, dry weight from 158.45 g to 319.67 g, longitudinal diameter increases to 47.22 mm, and transverse diameter expands to 20.33 mm. From 150 DAF to 180 DAF is the maturity phase, where the fruit peel turns orange-red and the flesh changes from orange-red to deep red. The size changes are not significant. At 180 DAF, the fresh weight and dry weight are 930.17 g and 329.17 g, respectively, with the longitudinal diameter and transverse diameter being 47.43 mm and 20.73 mm, respectively (**Figures 1B, C**).

### 2.2 Dynamic changes in oil content and fatty acid composition

**Figure 2:**
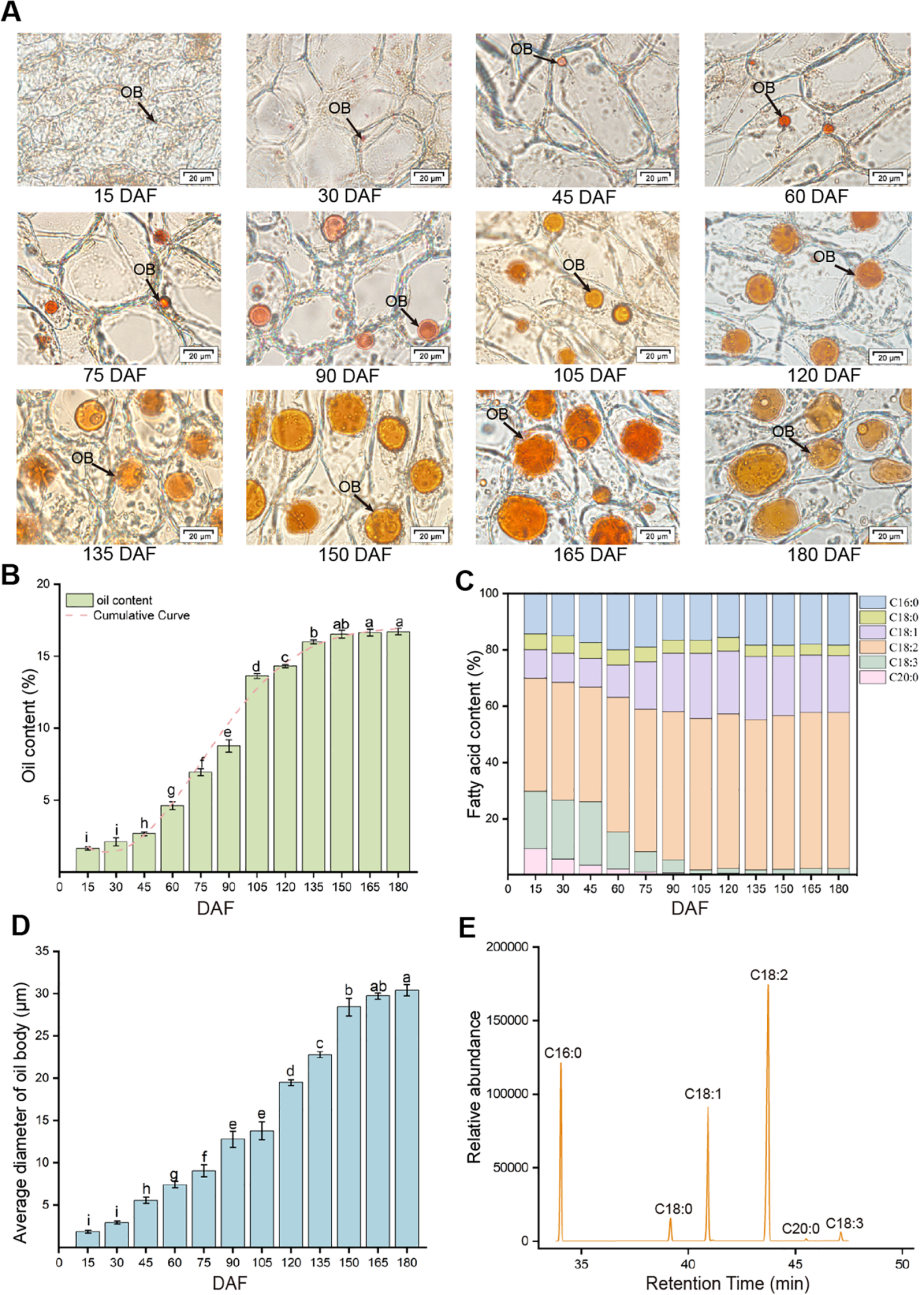
Dynamic Changes in oil content, fatty acids, and oil body morphology of *G. jasminoides* fruit. **(A)** Frozen sections of Gardenia fruit oil bodies; **(B)** Changes in oil content in *G. jasminoides*; **(C)** Fatty acid dynamics of *G. jasminoides* fruit at different developmental stages; **(D)** Diameter of *G. jasminoides* Fruit Oil Body; **(E)** Gas chromatogram of major fatty acids in mature Gardenia fruit.

To compare differences in key indicators such as oil content and oil body (OB) diameter across different developmental stages, a one-way analysis of variance (ANOVA) was employed, provided that the data met the assumptions of normality and homogeneity of variances (P<0.05). The accumulation pattern of *G. jasminoides* fruit oil follows an “S”-shaped curve and can be divided into four stages. During the initiation phase of oil synthesis (15~45 DAF), oil accumulated slowly, with oil content rising from an initial 1.6% to 2.7%. This is followed by the rapid accumulation phase (45~105 DAF), during which the oil content increased sharply from 2.7% to 13.6%. In the decelerated accumulation phase (105~150 DAF), oil content reached 16.5% at 150 DAF. Finally, in the stable accumulation phase (150~180 DAF), oil content remained relatively stable, peaking at 16.7% at 180 DAF. Based on changes in the shape, size, color, and oil content of the Gardenia fruit, it is concluded that the fruit reaches morphological and physiological maturity at 150 DAF (**Figure 2B**).

**Figure 3:**
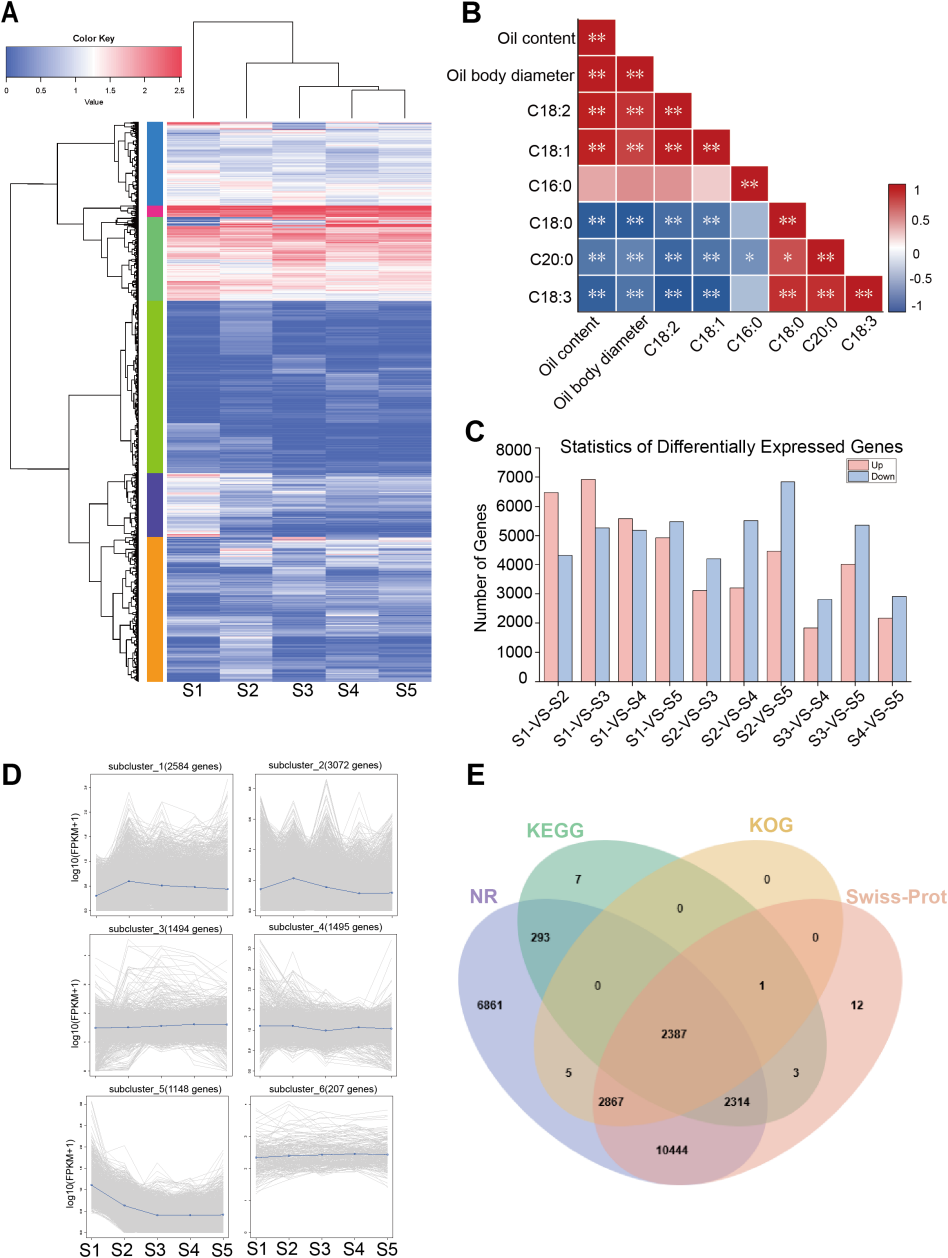
Analysis of DEGs in *G. jasminoides* fruit and correlation analysis with oil: **(A)** Hierarchical clustering diagram of DEGs; **(B)** Correlation analysis of oil content, oil body diameter, and major fatty acid content in *G. jasminoides* fruit(** *P* < 0.01, * *P <* 0.05, two-tailed); **(C)** statistics of up- and down-regulated DEGs in the transcriptome of *G. jasminoides* fruit; **(D)** Six distinct expression patterns; **(E)** Comparative Venn diagram analysis of unigene functional annotation across multiple public protein databases.

Through gas chromatography (GC) analysis of Gardenia fruits at different stages, six major fatty acids were identified: linoleic acid (C18:2), oleic acid (C18:1), palmitic acid (C16:0), stearic acid (C18:0), arachidic acid (C20:0), and linolenic acid (C18:3). Unsaturated fatty acids predominated, accounting for 77.6% of the total at maturity. C18:2 was the most abundant, averaging 55.2% at maturity, followed by C18:1 at 20.6%. To assess the associations between traits, bivariate correlation analysis was performed using the Pearson correlation coefficient (**Figure 3B**). Their levels increased during fruit development and then plateaued, showing significant positive correlations with both oil content, the correlation coefficients were 0.92 and 0.93, respectively. C16:0 followed, with a mean relative content of 18.3% at maturation. C18:0, C18:3, and C20:0 maintained a dynamic equilibrium after 120 DAF, with average relative contents of 0.4%, 3.8%, and 1.8% at maturity, respectively (**Figures 2C, E**).

### 2.3 Oil body observation

During the development of Gardenia fruit, the distribution, morphology, size, and quantity of oil bodies exhibit significant and regular changes. Under electron microscopy, oil bodies stained orange-red by Sudan III show an increase in number, enlargement in size, and a shift from sparse to dense distribution. Oil bodies begin forming between 15 DAF and 30 DAF, but their quantity remains low, and they are small in size at this stage. By 45 DAF, oil bodies become clearly visible with regular shapes, primarily located around the cell walls, with relatively large gaps between them. From 60 DAF to 150 DAF, as the fruit develops, the number of oil bodies around cell walls increases, their volume expands significantly, and their distribution shifts from the periphery of the cell walls toward the cell center, with spaces becoming narrower. Between 150 DAF and 180 DAF, there are no significant changes in the quantity or distribution of oil bodies, with only minor variations in their morphology and size (**Figures 2A**). During Gardenia fruit development, the diameter of oil body exhibited a continuous increase, reaching a maximum diameter of 29.8 μm (**Figure 2D**). The diameter of oil bodies exhibited the same developmental trend as oil content and the relative contents of C18:1 and C18:2, showing strong positive correlations with correlation coefficients of 0.95, 0.77, and 0.84, respectively. This indicates that, during fruit development, oil content in Gardenia is primarily influenced by oil bodies and is strongly associated with oil body size (**Figure 3B**).

### 2.4 RNA-seq and sequence alignment

To investigate the gene expression changes and underlying molecular mechanisms during the development of *G. jasminoides* fruits, transcriptome sequencing was performed on the fruit at five key developmental stages: 15 DAF (S1), 45 DAF (S2), 75 DAF (S3), 105 DAF (S4), and 150 DAF (S5). Three independent biological replicates were performed for each stage. This selection was based on comprehensive analyses of fruit morphology (**Figure 1A**) and oil content (**Figure 2B**). According to the fruit development process, fruit volume increased rapidly from 15 to 75 DAF, fruit color changed significantly from 75 to 150 DAF, and both fruit size and color stabilized from 150 to 180 DAF (**Figure 1A**). Combined with the dynamic changes in oil accumulation, the oil synthesis rate was relatively low from 15 to 45 DAF, increased markedly from 45 to 75 DAF, and entered a rapid accumulation phase from 75 to 105 DAF. From 150 to 180 DAF, oil accumulation stabilized, indicating the onset of fruit maturation (**Figure 2B**). In summary, the five selected key time points correspond to critical stages of fruit development and oil accumulation. After sequencing, the raw data were strictly filtered using fastp software, producing 20,719,307 clean reads (6,215,792,060 bp). After filtering, the GC content was 44.94%, and the Q20 and Q30 values were 98.37% and 95.48%, respectively, indicating that the transcriptome sequencing data were of high quality (**Table 1**).

**Table 1:** Statistics of raw data and clean data.

Category	Value
CleanData	20719307
CleanDatas(bp)	6215792060
Q20(%)	98.37
Q30(%)	95.48
GC(%)	44.94

### 2.5 Functional annotation and classification

To annotate and identify the specific functions of genes, all assembled unigenes were subjected to annotation using the Basic Local Alignment Search Tool (BLAST) against the publicly available protein databases. A total of 137,126 genes were annotated across all databases. Specifically, 100,491, 78,231, 46,505, and 27,695 unigenes were identified through BLAST searches against the Nr, SwissProt, KEGG, and KOG databases, respectively. The Nr database provided the highest number of annotated genes, accounting for 73.28% of all annotations (**Figure 3E**).

**Figure 4:**
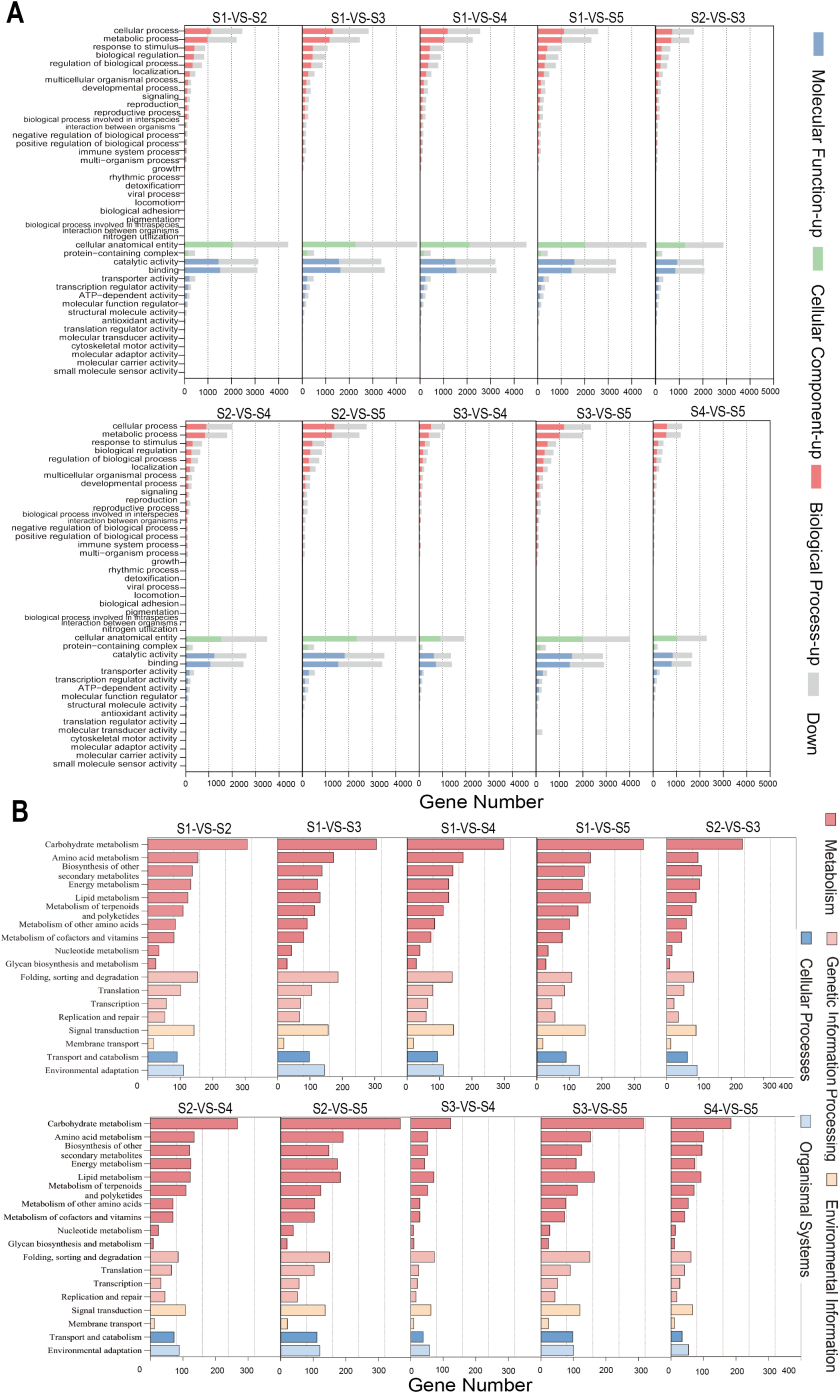
GO and KEGG annotation of DEGs in the transcriptome of *G. jasminoides* fruits: **(A)** GO functional classification classification of DEGs in *G. jasminoides* fruits; **(B)** Enrichment of KEGG metabolic pathway of DEGs in *G. jasminoides* fruits.

GO annotation results revealed that among the three major functional categories, Biological Process (BP) contained the highest number of annotated genes, followed by Cellular Component (CC) and Molecular Function (MF). BP was divided into 25 subcategories, among which cellular process (8,638 unigenes) and metabolic process (7,262 unigenes) were the most enriched. MF comprised 14 subcategories, predominantly represented by binding (10,230) and catalytic activity (9,316). CC was classified into only two subcategories, with the vast majority of unigenes annotated to cellular anatomical entity (13,878), followed by protein-containing complexes (2,046) (**Figure 4A**).

Based on the KEGG database annotation of the sequencing data, a total of 19,441 genes were successfully annotated. The KEGG Level A classification comprised five major categories, among which Metabolism contained the highest number of annotated genes (16,616), encompassing 10 Level B sub-pathways. Additionally, both Cellular Processes and Organismal Systems in Level A contained only one subcategory each at Level B. Among all annotated genes, 391 genes (accounting for 2.01%) were assigned to lipid metabolism-related pathways (**Figure 4B**).

### 2.6 Differentially expressed gene analysis

Gene expression levels for the *G. jasminoides* fruit transcriptome were quantified and normalized using RSEM to generate FPKM values. Differential expression analysis across developmental time points was performed with DESeq2 to identify differentially expressed genes (DEGs), and the numbers of up- and down-regulated unigenes for each comparison were summarized (**Figure 3C**). The results showed that the majority of DEGs were down-regulated during fruit development, with generally consistent expression patterns across all five stages. The S1-vs-S3 comparison showed the highest number of DEGs (12,185), comprising 6,920 up-regulated and 5,265 down-regulated genes. In contrast, the S3-vs-S4 comparison displayed the fewest DEGs (4,632), including 1,829 up-regulated and 2,803 down-regulated genes.

Cluster analysis was performed on the FPKM values of all DEGs, which categorized them into six clusters with similar expression patterns. The results showed that Cluster 1 and Cluster 2 shared consistent expression trends, both exhibiting an initial increase followed by a decrease, with their log10(FPKM + 1) values peaking at stage S2. Clusters 3, 4, and 6 displayed a generally slow upward trend, whereas Cluster 5 showed an initial decline followed by stabilization, specifically characterized by a significant down-regulation in log10(FPKM + 1) values from S1 to S3, which then remained stable from S3 to S5 (**Figures 3A, D**).

Gene Ontology (GO) functional annotation was performed on the DEGs identified between S1, S2, S3, S4, and S5. The results showed that within the Biological Process category, cellular process and metabolic process contained the highest numbers of DEGs, with the greatest enrichment observed in the S1-vs-S3 comparison, comprising 2,826 and 2,452 DEGs, respectively. In the Cellular Component category, the most annotated term was cellular anatomical entity with 2,891 DEGs. In the Molecular Function category, catalytic activity had the highest number of both up- and down-regulated genes, totaling 3,519 DEGs (**Figure 4A**).

KEGG pathway annotation and enrichment analysis were performed on all DEGs. The results showed that these genes were annotated to five major Level A categories (Metabolism, Genetic Information Processing, Environmental Information Processing, Cellular Processes, and Organismal Systems) and their subordinate 18 Level B subcategories. Among these, the Carbohydrate metabolism pathway contained the highest number of enriched DEGs, with 368 DEGs identified in the S2-vs-S5 comparison alone. Further analysis revealed notable differences in the number of lipid-metabolism enriched DEGs across various developmental stage comparisons: S1-vs-S2, S1-vs-S3, S1-vs-S4, S1-vs-S5, S2-vs-S3, S2-vs-S4, S2-vs-S5, S3-vs-S4, S3-vs-S5, and S4-vs-S5 contained 124, 132, 129, 165, 92, 123, 184, 72, 165, and 93 enriched DEGs, respectively (**Figure 4B**).

### 2.7 Identification and expression profiles of key DEGs involved in fatty acid and TAG biosynthesis

By enriching all DEGs in the Gardenia fruit transcriptome through KEGG metabolic pathways, a total of 13 lipid metabolic pathways were significantly enriched. These pathways mainly include the Biosynthesis of unsaturated fatty acids (ko 01040), the Fatty acid biosynthesis (ko 00061), the Glycerophospholipid metabolism(ko 00564), the Glycerolipid metabolism (ko 00561), and the Fatty acid elongation (ko 00062), among others. In the comparison groups S1-vs-S2, S1-vs-S3, S1-vs-S4, S1-vs-S5, S2-vs-S3, S2-vs-S4, S2-vs-S5, S3-vs-S4, S3-vs-S5, and S4-vs-S5, a total of 165, 177, 173, 237, 129, 169, 249, 99, 227, and 134 DEGs were identified, respectively. Among them, 88, 104, 103, 162, 84, 113, 170, 54, 142, and 79 were up-regulated DEGs, while 77, 73, 70, 75, 45, 56, 79, 45, 85 and 55 were down-regulated DEGs.

**Figure 5:**
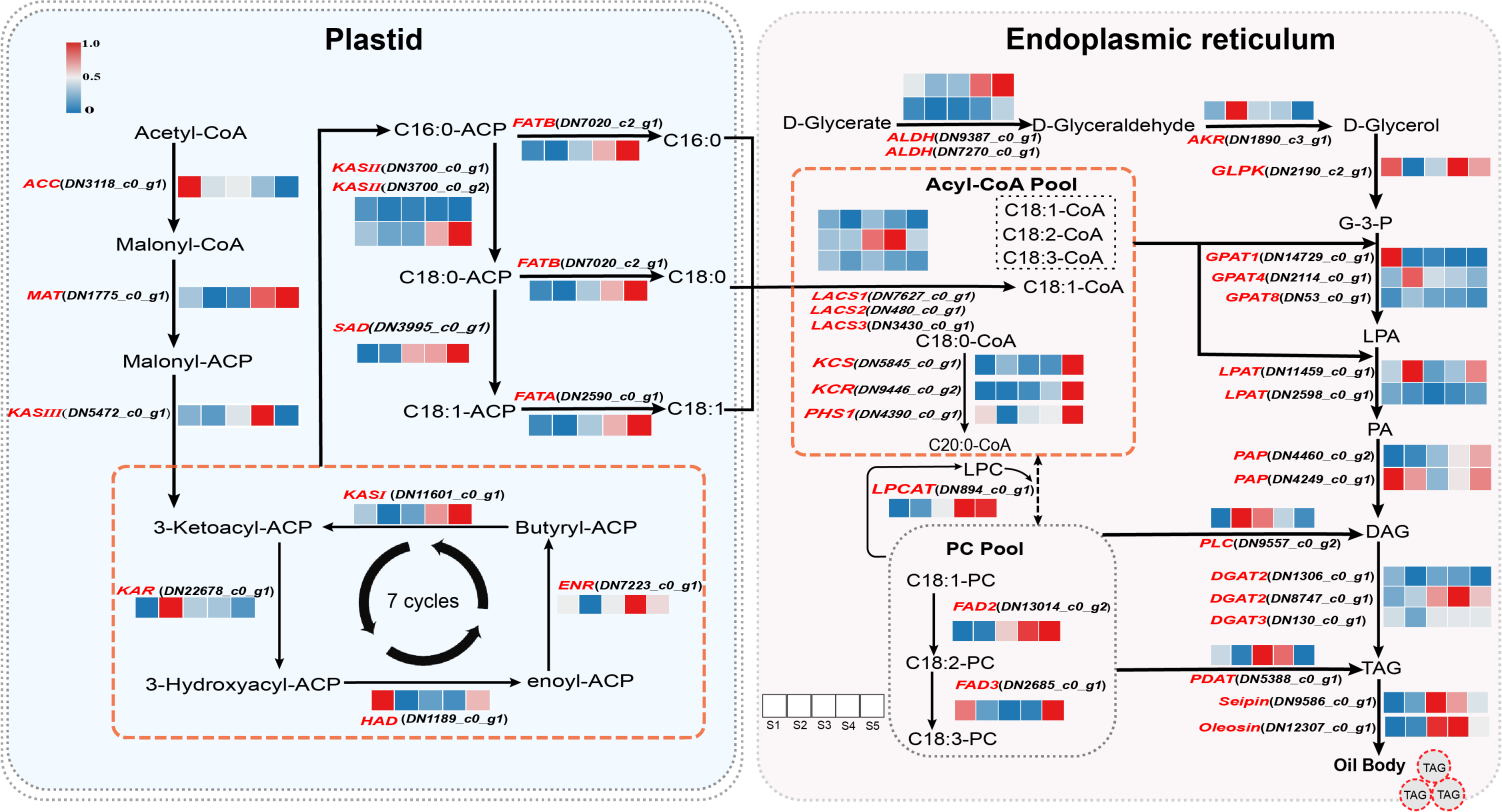
Schematic diagram of the lipid biosynthesis and metabolic pathways and their temporal expression patterns in *G. jasminoides* fruits. Note: acetyl-CoA carboxylase: ACC; Malonyl-CoA: ACP Transacylase: MAT; 3-oxoacyl-ACP synthase: KAS; 3-Ketoacyl-ACP Reductase: KAR; 3-Hydroxyacyl-ACP Dehydratase: HAD; Enoyl-ACP Reductase: ENR; acyl-ACP desaturase: SAD; fatty acyl-ACP thioesterase A: FATA; fatty acyl-ACP thioesterase B: FATB; long-chain acyl-CoA synthetase: LACS; 3-Ketoacyl-CoA Synthase: KCS; very-long-chain enoyl-CoA reductase: KCR; PASTICCINO 1: PHS1; ω-6 FA desaturase: FAD2; ω-3 FA desaturase: FAD3; acetaldehyde dehydrogenase: ALDH; alcohol dehydrogenase: AKR; glycerol kinase: GLPK; glycerol-3-phosphate acyltransferase: GPAT; Lysophosphatidic acid acyltransferase: LPAT; Phosphatidic Acid Phosphatase: PAP; diacylglycerol O-acyltransferase 3: DGAT3; diacylglycerol O-acyltransferase 2: DGAT2; phospholipid: diacylglycerol acyltransferase: PDAT; Lysophosphatidylcholine Acyltransferase: LPCAT; snGlycerol 3-phosphate: G-3-P; lysophosphatidic acids: LPA; Phosphatidic acid: PA; Diacylglycerol: DAG; Triacylglycerol: TAG; phosphatidylcholine: PC; Phosphoinositide-specific Phospholipase C: PLC; The vertically stacked blocks represent different genes encoding enzymes with this catalytic function, and the horizonal direction represents different stages.

A total of 6 transcription factors were screened in lipid metabolism, among which the *MYB_related1* transcription factor shows significant positive correlations with oil content and *FATA*, with correlation coefficients of 0.9254 and 0.93838, respectively (**Supplementary Figure 1**). Analysis of DEGs related to fatty acid biosynthesis, elongation, desaturation, and TAG assembly during the development of Gardenia fruits facilitates a more comprehensive understanding of the expression patterns of genes governing oil accumulation (**Figure 5**).

Oil biosynthesis can be divided into two core processes: fatty acid biosynthesis and TAG assembly. The first step in the fatty acid biosynthesis pathway is the conversion of acetyl-CoA to malonyl-CoA, the key step catalyzed by acetyl-CoA carboxylase (ACCase). The *ACC* gene is highly expressed during the S1~S3 but shows no significant expression during the S5. Among the FA synthase components, MAT catalyzes the conversion of malonyl-CoA to malonyl-ACP, and its gene expression is up-regulated during stages S4~S5. KAS III catalyzes the formation of acetoacetyl-ACP from malonyl-ACP, and the *KAS III* gene is up-regulated during the rapid oil synthesis phase (S3~S4). *KAS I*, *KAR*, and *ENR* are also up-regulated during the period of rapid oil accumulation (S3~S4). Compared with S1, most fatty acid biosynthesis-related DEGs exhibit higher FPKM values during the mid-developmental stages of the fruit, indicating that fatty acid biosynthesis is highly active at this time and drives the rapid accumulation of oil.

The acquisition of C18:0-ACP primarily relies on the catalytic synthesis by KASII enzyme, followed by further desaturation by acyl-ACP desaturase (SAD) enzyme to yield C18:1-ACP. *KASII* expression was up-regulated during S4 and S5 stages but down-regulated in S1 to S3 stages. *SAD* exhibited higher FPKM values during S3~S5 stages compared to earlier developmental phases. The relative proportion of C18:1 gradually increased starting from the S2 stage (**Figure 2C**), consistent with the significant expression of *SAD* during mid-development. C18:1 ranks second only to C18:2 in relative content within Gardenia fruit, with KASII and SAD enzymes playing pivotal roles. The elongation of very long-chain fatty acids (VLCFAs, ≥18 carbon atoms) is primarily regulated by enzymes such as 3-Ketoacyl-CoA Synthase (KCS), very-long-chain enoyl-CoA reductase (KCR), and PASTICCINO 1 (PHS1), which play a decisive role in the production of intermediates like C20:0-CoA. These enzyme genes showed up-regulated primarily during the S4~S5 stage, with *KASII* expression patterns aligning with its key role in catalyzing C18:0 synthesis. However, *KASII* exhibited higher FPKM values, indicating a more prominent contribution to enzymatic activity. This result also aligns with the fruit’s high C18:0 content and low C20:0 content.

Plant fatty acyl-ACP thioesterases (FATs) terminate the process of *de novo* fatty acid biosynthesis in plastids by hydrolyzing the acyl-ACP intermediates, and determine the chain length and levels of free fatty acids. FATs can release free fatty acids and ACP by hydrolyzing acylACP (Byers and Gong, 2007). FATA exhibits high substrate specificity toward monounsaturated acyl-ACPs and is primarily responsible for catalyzing the conversion of C18:1-ACP to C18:1 (Serrano-Vega et al., 2005; Sánchez-García et al., 2010; Moreno-Pérez et al., 2011). FATB predominantly mediates the release of saturated fatty acids and promotes their synthesis, primarily catalyzing the conversion of C16:0-ACP and C18:0-ACP into C16:0 and C18:0, respectively (Salas and Ohlrogge, 2002; Saha et al., 2006). FATA and FATB jointly regulate the saturation and carbon chain length distribution of plant oils. During Gardenia fruit development, their gene expression patterns are similar: *FATA* is down-regulated in stages S1 and S2, and up-regulated in stages S3~ S5, while *FATB* is up-regulated in stage S4~ S5. After fatty acid biosynthesis is completed, free fatty acids must be converted into fatty acyl-CoA to participate in subsequent chain elongation or TAG assembly. This activation process requires catalysis by LACS. The expression of different *LACS* homologs exhibits stage-specific patterns, but *LACS1, LACS2*, and *LACS3* all show elevated expression levels during the mid-fruit development stages S3 and S4. The expression of these *LACS* homologs provides a critical source of acyl-CoA substrates for TAG assembly.

C18:1 and C18:2 can undergo further desaturation to form polyunsaturated fatty acids with the help of PC. FAD2 catalyzes the desaturation of C18:1-PC to form C18:2-PC, with *FAD2* expression up-regulated during the S3~S5 stages. FAD3 catalyzes the desaturation of C18:2-PC to produce C18:3-PC, and *FAD3* shows higher FPKM values during the S1 stages, which aligns with the higher accumulation of C18:3 observed during these phases. Lysophosphatidylcholine Acyltransferase (LPCAT) plays a crucial role in the substrate cycling of C18:2 and C18:3 formation. LPCAT catalyzes the exchange between CoA and PC, particularly converting C18:1-CoA into C18:1-PC, thus providing the necessary phospholipid carrier for the continuous desaturation reactions of FAD2 and FAD3. *LPCAT* displayed higher FPKM values at stages S3~S5 (**Figure 5**).

Most fatty acids exist as TAGs or phosphoglycerides, with acylglycerols synthesized from two precursors: fatty acyl-CoA and G-3-P. During G-3-P synthesis, enzymes such as acetaldehyde dehydrogenase (ALDH), 3-Ketoacyl-ACP Reductase (AKR), and glycerol kinase (GLPK) are up-regulated during S2~S5 stages, providing an abundant supply of G-3-P substrates for the rapid lipid accumulation phase. Subsequently, GPAT converts G-3-P into LPA. *GPAT* homologs (*GPAT1*, *GPAT4*, *GPAT8*) display distinct expression patterns, with higher expression levels during the S1~S3 stages compared to S4 and S5. These *GPAT* homologs collectively initiate lipid accumulation.

Subsequently, LPAT synthesizes PA using LPA as a substrate. *LPAT* expression levels are higher during stages S1~S2 compared to stages S3~S5. PA is hydrolyzed by PAP into Diacylglycerol (DAG), with PAP exhibiting high expression throughout development. DAG undergoes TAG assembly via two parallel pathways, which synergistically optimize oil synthesis efficiency in Gardenia fruit through temporal coordination (Zhang et al., 2009). DG*AT* (*DGAT2*, *DGAT*3) homologs catalyze the condensation of DAG with Acyl-CoA to form TAG. These *DGAT* homologs exhibit sustained high expression during the rapid oil accumulation phase (S3~S5). PDAT pathway: PDAT mediates the transfer of phospholipid acyl groups to DAG, resulting in the formation of TAG and lysophospholipids. *PDAT* expression is significantly upregulated during the rapid oil accumulation phase (S3~S4).TAG is stored in specialized organelles known as oil bodies. Under the regulation of specific proteins, TAG buds off from the endoplasmic reticulum to form independent oil bodies, which are composed of TAG, phospholipids (PL), and intrinsic proteins. The intrinsic proteins include oleosin, caleosin, steroleosin, and lipid droplet-associated proteins (LDAPs), among others (Huang, 2018). Screening for oil body synthesis-related genes in Gardenia fruit identified two differentially expressed genes: *Seipin 2* and *Oleosin*. Both genes were significantly expressed during the stage when oil body diameter increased rapidly. *Seipin 2* and *Oleosin* were highly expressed from stage S3 to S5, coinciding with the period of rapid oil body expansion. The expression patterns of *Seipin 2* and *Oleosin* genes align well with the observed changes in oil body volume.

### 2.8 qRT-PCR

**Figure 6:**
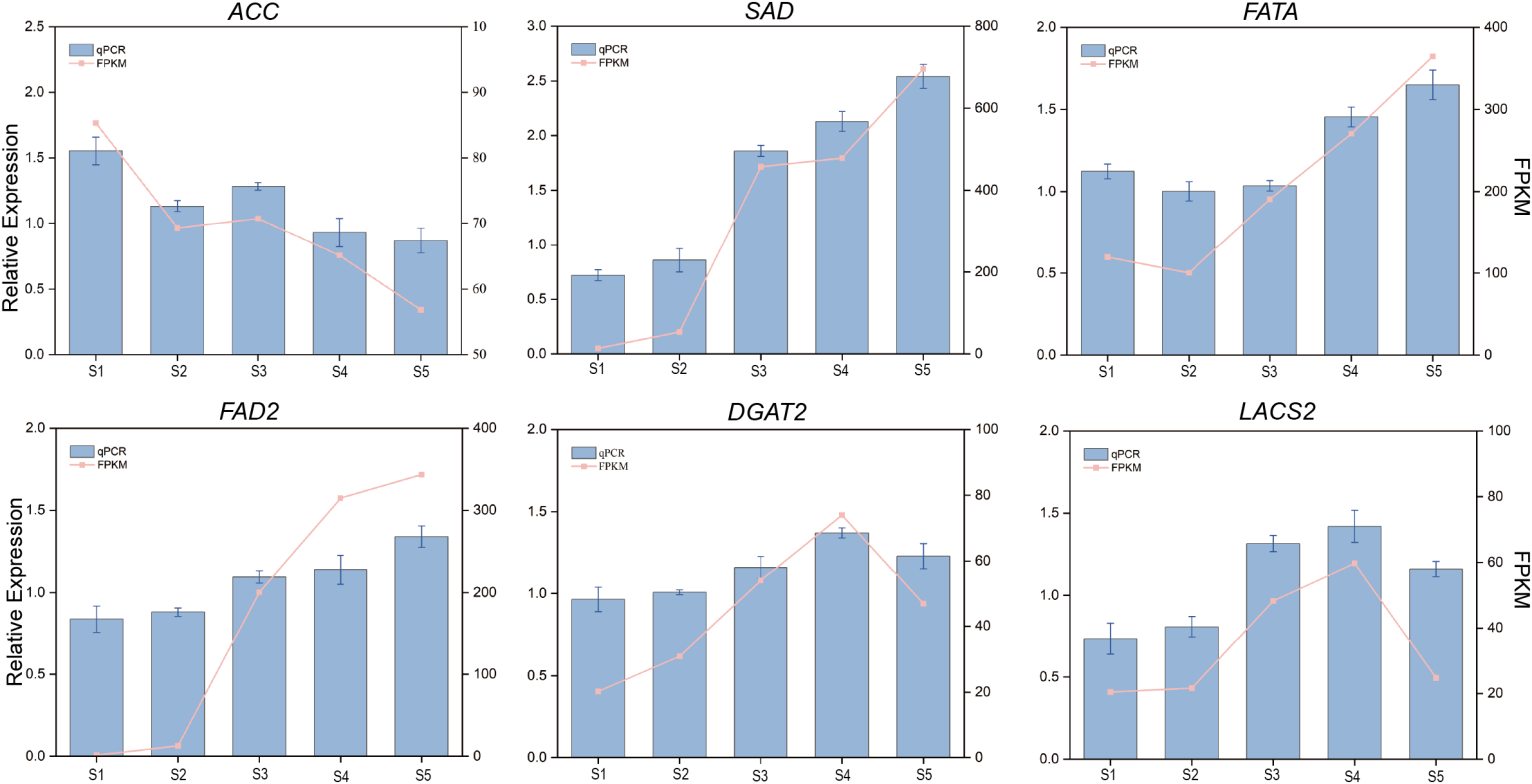
qRT-PCR validation of six candidate genes associated with oil biosynthesis during *G. jasminoides* fruits development.

To further validate the accuracy of the transcriptome sequencing results in *G. jasminoides* fruits six lipid-metabolism-related DEGs (*ACC*, *SAD*, *FATA*, *FAD2*, *DGAT2*, and *LACS2*) were selected for qRT-PCR analysis. 18S rRNA was used as the internal reference gene for quantification. The results showed that the gene expression patterns obtained by qRT-PCR were consistent with those from the RNA-seq data. These findings confirm the reliability of the transcriptome expression profiles and demonstrate that RNA-seq is suitable for detecting DEGs in *G. jasminoides* fruits (**Figure 6**).

## 3 Discussion

### 3.1 Oil content and fatty acid composition in gardenia fruits

Plant oil is composed of fatty acids and glycerol, among which fatty acids are the main components of oil, and their composition and content can be used as important indicators for evaluating oil crops (Absalome et al., 2020). The oil content of Gardenia (16.7%) is comparable to that of soybean and is consistent with previous reports, which have described an oil content of approximately 20% (Jin et al., 2023).

Although the oil content of Gardenia fruit is lower than that of traditional woody oil crops such as camellia (approximately 44% (Li et al., 2022)) and olive (approximately 40% (Zhang et al., 2024)), its potential for comprehensive utilization as a medicinal and edible plant underscores the significant research value of its oil resources. Gardenia fruit oil exhibits a high proportion of unsaturated fatty acids, primarily composed of C18:1 (20.6%) and C18:2 (55.2%) at maturity, which collectively account for 75.8% of the total. Saturated fatty acids are predominantly represented by C16:0 (18.3%). Research indicates that 18-carbon unsaturated fatty acids constitute a significant component of plant oils such as *Camellia oleifera* oil, *Carya cathayensis* oil, and *Juglans regia* oil, with their regulatory mechanisms varying across plant species. Among these oils, Gardenia and Juglans oils exhibit the most similar fatty acid compositions. Their primary fatty acids are C18:2 (42.5~76%), followed by C18:1(9~39.2%), C18:3(2~19.2%), C16:0 (2.9~11.4%), and C18:0(0.6~2.5%) (Amaral et al., 2003; Gao et al., 2018).

Oil bodies are the key organelles for storing TAG in oilseed crops. Their morphological characteristics (such as size, number, and surface protein coverage) and spatial distribution patterns directly influence oil stability and final oil content (Murphy, 2001). Heneen (Heneen et al., 2008) et al. found that in Avena sativa endosperm development, large oil bodies are formed by the fusion of smaller oil bodies, and oil body size is positively correlated with oil content, a trend also observed in the anatomy of Gardenia fruit oil bodies. Liu (Liu et al., 2024) et al. studied sandalwood and found that small oil bodies initially accumulate within mesocarp cells, subsequently fusing to form larger oil bodies. This process results in fewer changes in oil body number and size at maturity, consistent with the observation that oil body morphology and quantity remain stable at the maturity stage of Gardenia fruit.

### 3.2 Key enzyme genes involved in fatty acid biosynthesis

In lipid metabolism, fatty acid biosynthesis is a critical physiological process, precisely regulated by multiple key enzyme genes. Gardenia fruit oil is dominated by unsaturated fatty acids, with a marked enrichment of C18:2 content, which is related to the coordinated regulation of various key enzyme genes involved in fatty acid biosynthesis. In plants, *de novo* fatty acid synthesis begins in plastids, where the ACC enzyme catalyzes the conversion of acetyl-CoA to malonyl-CoA. This reaction represents the first rate-limiting step for carbon flux entering the fatty acid synthesis pathway (Post-Beittenmiller et al., 1991; Konishi et al., 1996). The functional integrity of ACC is decisive for all subsequent steps. Research by Caroca et al. (2021) demonstrated that knocking out *accD* reduces ACC activity, impairs triacylglycerol synthesis, and significantly decreases seed oil content. Subsequently, after multiple rounds of carbon chain elongation, the desaturation status of the chain and the timing of its termination and release collectively determine the final fatty acid composition of the oil. The SAD enzyme catalyzes the conversion of C18:0-ACP to C18:1-ACP within plastids, introducing the first double bond, thereby establishing the foundation for oil unsaturation. Following this, FATA specifically hydrolyzes unsaturated acyl-ACPs (e.g., C18:1-ACP), transporting the newly synthesized fatty acids from plastids to the endoplasmic reticulum for subsequent assembly. FATA primarily hydrolyzes unsaturated acyl-ACPs (e.g., C18:1-ACP), while FATB mainly hydrolyzes medium- and long-chain saturated fatty acids (e.g., C16:0-ACP and C18:0-ACP). This characteristic directly influences the ratio of saturated to unsaturated fatty acids in Gardenia fruit oil. The higher expression abundance of *FATA* compared to *FATB* throughout the fruit development period preferentially releases and exports the unsaturated C18:1-ACP. In the Gardenia fruit of this study, the content of C18:1 (20.6%) in mature oil was significantly higher than that of C18:0 (3.8%) (**Figure 2C**). This trait is precisely the result of the synergistic action of the aforementioned key genes. The high expression of *SAD* and *FATA* during the rapid oil accumulation phase in Gardenia ensures an adequate supply of C18:1-ACP, which constitutes an important molecular basis for the richness of unsaturated fatty acids (especially C18:1) in Gardenia fruit oil.

The significant enrichment of C18:2 in gardenia fruit oil (reaching 55.4% at maturity) is attributed to the regulation of the fatty acid desaturation pathway. Observations revealed that the FPKM values of *FAD2*, which encodes ω-6 desaturase in gardenia fruit, were consistently and significantly higher throughout the developmental period than those of *FAD3*, which encodes ω-3 desaturase. This leads to the near-unidirectional conversion of C18:1-PC to C18:2-PC in the PC pool, affecting further conversion to C18:3-PC. The expression of *FAD2* coincides with the rising phase of C18:2, ensuring the synthesis of C18:2. FAD2 dominates the composition of polyunsaturated fatty acids, a pattern widely confirmed in traditional oil crops such as rapeseed (Wells et al., 2014). Gardenia exhibits distinct fatty acid composition characteristics. In this study, sustained high expression of *FAD2*, significantly exceeding that of *FAD3*, was observed. Through an extreme expression ratio of *FAD2* to *FAD3*, it achieves a more abundant C18:2 output compared to crops like rapeseed (Wang et al., 2015), which may be related to the medicinal functions of gardenia. Gardenia fruit is similar to Qinghai walnuts, both being predominantly composed of eighteen-carbon unsaturated fatty acids. This study further found that the expression of the SAD and FAD2 genes was significantly up-regulated during the rapid oil accumulation phase in gardenia fruit, with markedly increased FPKM values, which is consistent with observations from studies on Qinghai walnuts (Shi et al., 2022). These results further indicate that SAD and FAD2 are important molecular foundations for the richness of unsaturated fatty acids in oils. Therefore, from *ACC* (initiating synthesis), *SAD* (determining initial unsaturation), *FATA* (regulating output composition) to *FAD2* (achieving high unsaturation), the coordinated regulation of these genes effectively drives the formation of the unique high-unsaturated fatty acid phenotype in gardenia fruit oil, resulting in a characteristic fatty acid profile dominated by C18:2.

### 3.3 Key enzyme genes involved in TAG biosynthesis in gardenia fruits

TAG serves as the primary storage lipid in mature seeds and represents the final product assembled through multiple enzymatic reactions involving fatty acids and a glycerol backbone (Zhu et al., 2012). TAG biosynthesis originates from two core substrates: G-3-P and fatty acyl-CoA. G-3-P is supplied via two distinct pathways: the reduction of dihydroxyacetone phosphate and the phosphorylation of glycerol catalyzed by GLPK. Fatty acyl-CoA is generated through the activation of free fatty acids catalyzed by LACS. LACS belongs to the carboxyl-CoA ligase superfamily. Its primary function is to activate fatty acids to form fatty acyl-CoAs, thereby providing activated acyl substrates for all subsequent lipid synthesis reactions; thus, LACS is indispensable for TAG production. In this study, we observed that the FPKM value of *LACS2* was significantly higher than that of other LACS homologs, indicating that LACS2 is likely the major contributor to the fatty acid activation step. We therefore propose that *LACS2* is a key enzymatic gene in the TAG biosynthetic pathway in gardenia fruit.

TAG assembly occurs in the endoplasmic reticulum and is coordinately accomplished through both the acyl-CoA-dependent pathway (Kennedy pathway) and the acyl-CoA-independent pathway (non-Kennedy pathway). The key distinction between these two pathways lies in the mechanism of diacylglycerol (DAG) conversion to TAG during the final step of TAG synthesis.In the Kennedy pathway, DGAT, as the key final enzyme, catalyzes the conjugation of acyl-CoA with DAG to form TAG. In the Kennedy pathway, PDAT plays the central role by directly transferring an acyl group from PC to DAG, thereby completing TAG synthesis. These two pathways function complementarily in plant oil biosynthesis, working synergistically to regulate the efficiency of TAG production (Zhao et al., 2018). The differential gene expression analysis in gardenia fruit revealed that the FPKM value of *DGAT* was significantly higher than that of *PDAT*, suggesting that DGAT plays a key role in TAG synthesis and that the Kennedy pathway predominates in TAG biosynthesis in this species. Research by Jin et al (Jin et al., 2020). demonstrated that overexpression of the *EgDGAT2* gene in *Arabidopsis thaliana* seeds increased the content of polyunsaturated fatty acids such as C18:2 and C18:3 in seed TAG, indicating that DGAT2 exhibits a catalytic preference for esterifying unsaturated fatty acids (e.g., C18:2). Fatty acid composition analysis of gardenia fruit (**Figure 2C**) showed that the oil of mature fruits is predominantly composed of unsaturated fatty acids, with C18:2 content as high as 55.4%. Given the catalytic preference of DGAT2 for unsaturated fatty acids, DGAT2 is considered a key enzymatic gene for TAG synthesis in gardenia fruit. Furthermore, oil accumulation analysis in this study (**Figure 2B**) revealed that the period of rapid oil accumulation in gardenia fruit coincided significantly with the high expression of both *LACS2* and *DGAT2* genes, identifying these two genes at the transcriptional level as key candidates for TAG synthesis in gardenia fruit. The key genes (such as *FATA*, *SAD*, *FAD2*) screened in this study show significant positive correlations with the *MYB_related1*, suggesting that they may be coordinately regulated by common upstream factors. Whether the *MYB_related1* controls oil accumulation in the gardenia fruit investigated in this study warrants further validation. It is noteworthy that existing studies have reported the regulatory role of similar transcription factors in lipid accumulation. Cao et al. (Cao et al., 2023) identified the *R2 R3-MYB* transcription factors in the Euphorbiaceae family, and found that overexpressing *VfMYB36* significantly increases seed oil content. This indicates that *MYB36* is a positive regulator of oil accumulation.

In plant cells, TAG is synthesized in the endoplasmic reticulum and stored in specialized organelles called oil bodies through a budding process. Oil bodies are primarily composed of TAG, PL, and intrinsic proteins (Hsieh and Huang, 2004; Shao et al., 2019). Both Seipin and Oleosin are crucial proteins in the plant oil storage system and cooperatively regulate oil body biogenesis, yet they perform distinct functions. Seipin proteins are localized at the ER-oil body junctions, where they mediate the directional formation of oil bodies into the cytoplasm and ensure proper budding of oil bodies from the ER (Greer et al., 2020). Seipin proteins promote the accumulation of oil bodies (Cai et al., 2015). During oil body formation in Gardenia fruits, the expression of *Seipin* showed a significant positive correlation with oil body diameter, indicating its important role in oil body synthesis in this species. Oleosin is a key structural component of the oil body membrane, involved not only in the formation and stabilization of oil bodies but also playing a critical role in lipid storage (Huang, 2018). The results showed that the expression of the *Oleosin* gene in Gardenia fruit was continuously up-regulated from stages S3 to S5, consistent with the rapid expansion of oil body volume during this period, suggesting that it may participate in oil body biosynthesis and structural maintenance during lipid storage in Gardenia fruit.

## 4 Conclusion

This study systematically elucidated the patterns and molecular basis of oil accumulation in Gardenia jasminoides fruits through the integration of physiological, biochemical, and transcriptomic analyses. The development of Gardenia fruits was divided into three stages: fruit expansion period (15~60 DAF), color transition period (60~150 DAF), and maturation period (150~180 DAF). The oil accumulation in Gardenia fruits exhibits a characteristic “S”-shaped growth curve, consisting of the initial accumulation phase (15~45 DAF), rapid accumulation phase (45~105 DAF), decelerated accumulation phase (105~150 DAF), and stabilization phase (150~180 DAF). At 150 DAF, Gardenia fruits achieved both morphological and physiological maturity, with the entire maturation process requiring 150 days, providing critical theoretical guidance for the optimal harvesting time of Gardenia fruits. The study revealed the high unsaturation characteristics of Gardenia oil. The main fatty acids in mature fruits were C18:1 and C18:2, which stabilized after 135 DAF, with average relative contents of 20.6% and 55.2%, respectively, at maturity. The diameter of oil bodies exhibits a patterned increase during fruit development, expanding rapidly from 15 to 150 DAF and remaining relatively stable from 150 to 180 DAF. Correlation analysis indicated that the diameter of oil bodies showed a significant positive correlation with the developmental process of gardenia fruit and oil accumulation, indicating that the enlargement of oil bodies is a key structural feature for lipid storage. Finally, through the analysis of dynamic changes in fatty acids and gene expression, *ACC*, *SAD*, *FATA*, and *FAD2* were screened as candidate key genes contributing to the high proportion of C18:2, while *LACS2* and *DGAT2* were screened as key candidate genes involved in TAG synthesis in Gardenia fruits.

## 5 Materials and methods

### 5.1 Experimental materials

The experimental materials were collected from the Gardenia planting base of Hunan Hi-tech Bio-agro Co., Ltd. (113.004789°E, 28.859502°N), which belongs to the subtropical monsoon climate. Three healthy, pest-free, and uniformly growing 5-year-old Gardenia plants were selected. Every 15 days, 90 fruits were collected and thoroughly mixed, then divided into three portions from 15 to 180 DAF. Intact, healthy fruits were gathered from all four cardinal directions (east, west, south, and north) of each plant. One portion of the samples was flash-frozen in liquid nitrogen for over 20 min, temporarily stored on dry ice, and subsequently transferred to a -80 °C ultra-low temperature freezer in the laboratory. Another portion was preserved in Formalin-Aceto-Alcohol (FAA) fixative solution specifically for oil body observation.

Fifteen fresh fruits were randomly selected and divided into 5 groups (with three fruits per group, randomly assigned) to observe and measure their morphological indices, thereby assessing phenotypic traits during fruit development. Another 15 fresh fruits were randomly chosen and placed in an oven (dried at 70 °C for 72 hours). They were then stored in a cool and dry place for subsequent subsequent experiments (determination of oil content and fatty acid composition) (n=3).

### 5.2 Fruit phenotyping

Measure the fruit length and width of each plant using a vernier caliper, repeating the measurement five times. Determine the hundred-fruit weight of fresh fruit using an FA2004B electronic analytical balance, repeating the measurement five times.

### 5.3 Oil body observation

Observation of oil bodies in Gardenia fruit using the Frozen Section Method, following the procedure described by Liu (Liu et al., 2024) et al. Fruits previously fixed in FAA solution were retrieved from a 4°C refrigerator and repeatedly rinsed with distilled water until all residual fixative was completely removed. The surface moisture was blotted dry with filter paper, after which the samples were immersed in a glycerol solution. The specimens were then placed in a glass vacuum desiccator, and vacuum infiltration was carried out using a vacuum pump to ensure thorough penetration of glycerol into the intercellular spaces of the tissue. The cryostat (M205C, Leica, Germany) was set to -30°C for sectioning. The samples were rapidly frozen for 30 seconds and obtain 25 mm thick sections as temporary slides. These slides were stained with Sudan III solution for 20 minutes. Finally, the slides were observed and photographed using an electron microscope system.

### 5.4 Oil content determination

The oil content of the Gardenia fruit was determined following the Soxhlet extraction method outlined in the Chinese standard Determination of Fat in Foods (GB 5009.6-2016) (State Food and Drug Administration, 2016). After cleaning, the Gardenia fruits were dried in an oven until they reached a constant weight (75°C, 24 hours), then pulverized. A sample weighing 3.0~5.0 g (denoted as M_0_) was taken and extracted using petroleum ether (99.7%, boiling range 30~60 °C) as the solvent in a Soxhlet extractor. The extraction was conducted with 100 mL of petroleum ether by reflux for 8 hours. After extraction, the sample was removed, dried, and placed in a 75°C oven until it reached a constant weight. Once cooled to room temperature in a desiccator, the sample was weighed again, and the final weight (M_1_) was recorded. The oil content W (accurate to 0.0001 g) was then calculated using the following formula: W = (M_0_ − M_1_)/M_0_ × 100%.

### 5.5 Gas chromatography analysis

The determination of FA methyl esters and content was conducted following the GB 5009.6-2016 method (State Food and Drug Administration, 2016). For FA methyl ester analysis, weigh approximately 0.06 g of Gardenia fruit oil sample, mix with 4 mL of isooctane and 200 µL of potassium hydroxide-methanol solution (2 mol/L) to dissolve the oil, shake the mixture thoroughly for 60 seconds, and allow it to settle for 30 minutes. Then, add 1.0 g of anhydrous NaHSO_4_;, collect the supernatant, filter it through a 0.22 μm organic filter membrane, and proceed with the determination. The content of Gardenia is determined using gas chromatography.

The relative contents of fatty acids in *Gardenia jasminoides* fruits were determined using a gas chromatograph (Shimadzu Nexis GC-2030, Shimadzu, Kyoto, Japan). The gas chromatography operating parameters were set according to the method described by Zhou (Zhou X. et al., 2024) et al.

### 5.6 RNA-seq and gene annotation

Transcriptome sequencing was performed on Gardenia fruits collected at 15 DAF (S1), 45 DAF (S2), 75 DAF (S3), 105 DAF (S4) and 150 DAF (S5). Three independent biological replicates were established for Gardenia fruit samples at each key developmental stage (S1~S5). For each biological replicate, four fruits were collected from different orientations of the plant, uniformly ground, and thoroughly mixed to create a composite sample. After homogenizing the whole fruits into a uniform powder, total RNA was subsequently extracted from each replicate using the Trizol reagent kit (Invitrogen, Carlsbad, CA, USA). RNA quality was assessed using an Agilent 2100 Bioanalyzer (Agilent Technologies, Palo Alto, CA, USA). After extracting total RNA, rRNA was removed and mRNA was enriched. The enriched mRNA was then fragmented using fragmentation buffer. The Illumina NEBNext Ultra RNA Library Preparation Kit (NEB #7530, New England Biolabs, Ipswich, MA, USA) was used to prepare the library. Reverse transcription was performed using a reverse transcription kit to convert the mRNA into a cDNA library, which was then sequenced.

Quality control of raw sequencing data was performed using fastp, which filtered out low-quality data to obtain high-quality data. Sequence assembly was performed using Trinity software, which assembled all clean reads into unigenes (Chen et al., 2018). The unigenes were aligned with multiple public protein databases using the Basic Local Alignment Search Tool (BLAST), including the NCBI Non-Redundant Protein Database (NR), the Swiss-Prot Database (SwissProt), the Gene Ontology Database (GO), the Co-Organism Group Database (COG), and the Kyoto Encyclopedia of Genes and Genomes (KEGG), with an E-value threshold set at 10^−5^ (Altschul et al., 1997). Gene differential expression analysis was performed using DESeq 2 (Love et al., 2014) software, with screening criteria of FDR < 0.05, log_2_FC > 1, or log_2_FC < -1. DEGs were functionally annotated in the GO and KEGG databases. RSEM (Li and Dewey, 2011) software was used to obtain gene expression levels, with Bowtie2 called for alignment.

### 5.7 qRT-PCR

Six key genes, *ACC, SAD, FATA, FAD2, DGAT2* and *LACS2*, related to the oil biosynthesis pathway, were selected for validation. Primers were designed using Premier 5.0 software (Premier Biosoft International, Palo Alto, CA, USA), with the 18S rRNA (**Table 2**) serving as the internal reference gene for quantitative analysis (Premier Biosoft International, Palo Alto, CA, USA). The qT-PCR reaction conditions for the samples included an initial denaturation step at 95°C for 3 minutes, followed by 40 amplification cycles. Each cycle included a 15-second denaturation step at 95°C, a 15-second annealing step, and a 30-second extension step at 60°C. Finally, the samples were denatured at 95°C for 15 s denaturation step, and each reaction was repeated three times. The relative expression levels of each gene were determined using the comparative cycle threshold method (Schmittgen and Livak, 2008).

**Table 2:** qRT-PCR gene primers sequence for RNA-seq validation in *G. jasminoides* fruits.

Number	Gene ID	Gene name	Gene primers
1	DN3118_ c0_ g1	*ACC*	F: GGCCACAAAGAAATGAGGGA R: CAGCATAGCAGCACAACAAAAC
2	DN3995_ c0_ g1	*SAD*	F: TCTTTCTTTCTGGAAGGGTGG R: CTCCTGGAACGAGGTGTAAATG
3	DN2590_ c0_ g1	*FATA*	F: CCGGTTCTGGCTGTGGTTA R: CGGTGGCAGTCTTGTTGATT
4	DN13014_ c0_ g2	*FAD2*	F: TCAAGAAAGTCATCCCACCTCA R: ATCCCTGGAAAACCCAATACA
5	DN8747_ c0_ g1	*DGAT2*	F: ATGGCGACGATGGAGATGA R: AAGGAGGCGAAGACGACAAG
6	DN480_ c0_ g1	*LACS2*	F: CTTTTCTTGGGATGAGTTTGCT R: CGCCTTTTGGTTCTCCTGTT

## Data Availability

The data presented in this study are available within the article and **Supplementary Materials**. The G. jasminoides fruits transcriptome raw data can be obtained from the China National Center for Bioinformation (https://ngdc.cncb.ac.cn), GSA number is PRJCA051768.

## References

[B1] Absalome M. A. Massara C.-C. Alexandre A. A. Gervais K. Chantal G. G.-A. Ferdinand D. . (2020). Biochemical properties, nutritional values, health benefits and sustainability of palm oil. Biochimie 178, 81–95. doi: 10.1016/j.biochi.2020.09.019 32966855

[B2] Altschul S. F. Madden T. L. Schäffer A. A. Zhang J. Zhang Z. Miller W. . (1997). Gapped BLAST and PSI-BLAST: a new generation of protein database search programs. Nucleic Acids Res. 25, 3389–3402. doi: 10.1093/nar/25.17.3389 9254694 PMC146917

[B3] Amaral J. S. Casal S. Pereira J. A. Seabra R. M. Oliveira B. P. P. (2003). Determination of sterol and fatty acid compositions, oxidative stability, and nutritional value of six walnut (*Juglans regia* L.) cultivars grown in Portugal. J. Agric. Food Chem. 51, 7698–7702. doi: 10.1021/jf030451d 14664531

[B4] Bates P. D. Stymne S. Ohlrogge J. (2013). Biochemical pathways in seed oil synthesis. Curr. Opin. Plant Biol. 16, 358–364. doi: 10.1016/j.pbi.2013.02.015 23529069

[B5] Bourgis F. Kilaru A. Cao X. Ngando-Ebongue G. F. Drira N. Ohlrogge J. B. . (2011). Comparative transcriptome and metabolite analysis of oil palm and date palm mesocarp that differ dramatically in carbon partitioning. Proc. Natl. Acad. Sci. U.S.A. 108, 12527–12532. doi: 10.1073/pnas.1106502108 21709233 PMC3145713

[B6] Byers D. M. Gong H. (2007). Acyl carrier protein: Structure-Function Relationships in a Conserved Multifunctional Protein Family. Biochem. Cell Biol. 85, 649–662. doi: 10.1139/o07-109 18059524

[B7] Cai Y. Goodman J. M. Pyc M. Mullen R. T. Dyer J. M. Chapman K. D. (2015). Arabidopsis SEIPIN proteins modulate triacylglycerol accumulation and influence lipid droplet proliferation. Plant Cell. 27, 2616–2636. doi: 10.1105/tpc.15.00588 26362606 PMC4815042

[B8] Cao Y. P. Fan T. T. Wang L. H. Zhang L. Li Y. L. (2023). Large-scale analysis of putative Euphorbiaceae R2R3-MYB transcription factors identifies a MYB involved in seed oil biosynthesis. Plant Physiol. 23, 145. doi: 10.1186/s12870-023-04163-5 36927311 PMC10022305

[B9] Caroca R. Howell K. A. Malinova I. Burgos A. Tiller N. Pellizzer T. . (2021). Knockdown of the plastid-encoded acetyl-CoA carboxylase gene uncovers functions in metabolism and development. Plant Physiol. 185, 1091–1110. doi: 10.1093/plphys/kiaa106 33793919 PMC8133629

[B10] Çelikel F. G. Reid M. S. Jiang C. Z. (2020). Postharvest physiology of cut *Gardenia jasminoides* flowers. Sci. Hortic. 261, e108983. doi: 10.1016/j.scienta.2019.108983 38826717

[B11] Chen L. Li M. Yang Z. Tao W. Wang P. Tian X. . (2020). *Gardenia jasminoides* Ellis: Ethnopharmacology, phytochemistry, and pharmacological and industrial applications of an important traditional Chinese medicine. J. Ethnopharmacol. 257, 112829. doi: 10.1016/j.jep.2020.112829 32311486

[B12] Chen Q. C. Youn U. Min B. S. Bae K. (2008). Pyronane monoterpenoids from the fruit of *Gardenia jasminoides*. J. Nat. Prod. 71, 995–999. doi: 10.1021/np800002z 18505286

[B13] Chen S. F. Zhou Y. Q. Chen Y. R. Gu J. (2018). Fastp: An ultra-fast all-in-one FASTQ preprocessor. Bioinformatics 34, i884–i890. doi: 10.1093/bioinformatics/bty560 30423086 PMC6129281

[B14] Chyau C. C. Chiu C. Y. Hsieh H. L. Hsieh D. W. C. Hsieh C. R. Chang C. H. . (2022). High-purity preparation of enzyme transformed transcrocetin reclaimed from Gardenia fruit waste. Plants 11, 281. doi: 10.3390/plants11030281 35161261 PMC8839004

[B15] Debnath T. Park P.-J. Deb Nath N. C. Samad N. B. Park H. W. Lim B. O. (2011). Antioxidant activity of *Gardenia jasminoides* Ellis fruit extracts. Food Chem. 128, 697–703. doi: 10.1016/j.foodchem.2011.03.090 38826717

[B16] Gao P. Jin J. Liu R. Jin Q. Wang X. (2018). Chemical compositions of walnut (*Juglans regia* L.) oils from different cultivated regions in China. J. Am. Oil Chem. Soc 95, 825–834. doi: 10.1002/aocs.12097 41531421

[B17] Gibellini F. Smith T. K. (2010). The Kennedy pathway—*De novo* synthesis of phosphatidylethanolamine and phosphatidylcholine. IUBMB Life. 62, 414–428. doi: 10.1002/iub.337 20503434

[B18] Greer M. S. Cai Y. Gidda S. K. Esnay N. Kretzschmar F. K. Seay D. . (2020). SEIPIN isoforms interact with the membrane-tethering protein VAP27-1 for lipid droplet formation. Plant Cell. 32, 2932–2950. doi: 10.1105/tpc.19.00771 32690719 PMC7474298

[B19] Heneen W. K. Karlsson G. Brismar K. Gummeson P.-O. Marttila S. Leonova S. . (2008). Fusion of oil bodies in endosperm of oat grains. Planta 228, 589–599. doi: 10.1007/s00425-008-0761-x 18563438

[B20] Hsieh K. Huang A. H. C. (2004). Endoplasmic reticulum, oleosins, and oils in seeds and tapetum cells. Plant Physiol. 136, 3427–3434. doi: 10.1104/pp.104.051060 15542496 PMC527141

[B21] Huang A. H. C. (2018). Plant lipid droplets and their associated proteins: Potential for rapid advances. Plant Physiol. 176, 1894–1918. doi: 10.1104/pp.17.01677 29269574 PMC5841732

[B22] Jin C. Wang L. Liu X. Lu Y. Yu N. Nie X. . (2023). Health oil preparation from Gardenia seeds by aqueous enzymatic extraction combined with puffing pretreatment and its properties analysis. Food Sci. Biotechnol. 32, 2043–2055. doi: 10.1007/s10068-023-01319-9 37860735 PMC10581964

[B23] Jin Y. H. Yuan Y. J. Gao L. C. Sun R. H. Chen L. Z. Li D. D. . (2020). Characterization and Functional Analysis of a Type 2 Diacylglycerol Acyltransferase (*DGAT2*) Gene from Oil Palm (*Elaeis guineensis* Jacq.) Mesocarp in *Saccharomyces cerevisiae* and Transgenic *Arabidopsis thaliana*. Front. Plant Sci. 11. doi: 10.3389/fpls.2017.01791 29089956 PMC5651047

[B24] Konishi T. Shinohara K. Yamada K. Sasaki Y. (1996). Acetyl-coA carboxylase in higher plants: most plants other than gramineae have both the prokaryotic and the eukaryotic forms of this enzyme. Plant Cell Physiol. 37, 117–122. doi: 10.1093/oxfordjournals.pcp.a028920 8665091

[B25] Koo A. J. K. Ohlrogge J. B. Pollard M. (2004). On the export of fatty acids from the chloroplast. J. Biol. Chem. 279, 16101–16110. doi: 10.1074/jbc.M313609200 14764601

[B26] Lee H. G. Seo P. J. (2019). Interaction of DGAT1 and PDAT1 to enhance TAG assembly in *Arabidopsis*. Plant Signal. Behav. 14, 1554467. doi: 10.1080/15592324.2018.1554467 30537885 PMC6351085

[B27] Li B. Dewey C. N. (2011). RSEM: accurate transcript quantification from RNA-Seq data with or without a reference genome. BMC Bioinf. 12, 323. doi: 10.1186/1471-2105-12-323 21816040 PMC3163565

[B28] Li N. Fan M. Li Y. Qian H. Zhang H. Qi X. . (2020). Stability assessment of crocetin and crocetin derivatives in Gardenia yellow pigment and Gardenia fruit pomace in presence of different cooking methods. Food Chem. 312, 126031. doi: 10.1016/j.foodchem.2019.126031 31874411

[B29] Li G. Ma L. Yan Z. Zhu Q. Cai J. Wang S. . (2022). Extraction of oils and phytochemicals from *Camellia oleifera* seeds: trends, challenges, and innovations. Processes 10, 1489. doi: 10.3390/pr10081489

[B30] Li-Beisson Y. Shorrosh B. Beisson F. Andersson M. X. Arondel V. Bates P. D. . (2013). Acyl-lipid metabolism. Arabidopsis Book. 11, e0161. doi: 10.1199/tab.0161 23505340 PMC3563272

[B31] Liu Q. Chen Y. Chen J. Li P. Jiang L. Li C. . (2024). Comparative analysis of transcriptome in oil biosynthesis between seeds and non-seed tissues of *Symplocos paniculata* fruit. Front. Plant Sci. 15. doi: 10.3389/fpls.2024.1441602 39416484 PMC11479902

[B32] Love M. I. Huber W. Anders S. (2014). Moderated estimation of fold change and dispersion for RNA-seq data with DESeq2. Genome Biol. 15, 550. doi: 10.1186/s13059-014-0550-8 25516281 PMC4302049

[B33] Moreno-Pérez A. J. Sanchez-Garcia A. Salas J. J. Garces R. Martinez-Force E. (2011). Acyl-ACP thioesterase from macadamia (*Macadamia tetraphylla*) nuts: cloning, characterization and their impact on oil composition. Plant Physiol. Biochem. 49, 82–87. doi: 10.1016/j.plaphy.2010.10.002 21071236

[B34] Murphy D. J. (2001). The biogenesis and functions of lipid bodies in animals, plants and microorganisms. Prog. Lipid Res. 40, 325–438. doi: 10.1016/S0163-7827(01)00013-3 11470496

[B35] Post-Beittenmiller D. Jaworski J. G. Ohlrogge J. B. (1991). *In vivo* pools of free and acylated acyl carrier proteins in spinach. Evidence for sites of regulation of fatty acid biosynthesis. J. Biol. Chem. 266, 1858–1865. doi: 10.1016/S0021-9258(18)52372-3 1988450

[B36] Saha S. Enugutti B. Rajakumari S. Rajasekharan R. (2006). Cytosolic triacylglycerol biosynthetic pathway in oilseeds. Molecular cloning and expression of peanut cytosolic diacylglycerol acyltransferase. Plant Physiol. 141, 1533–1543. doi: 10.1104/pp.106.082198 16798944 PMC1533943

[B37] Salas J. J. Ohlrogge J. B. (2002). Characterization of substrate specificity of plant FatA and FatB acyl-ACP thioesterases. Arch. Biochem. Biophys. 403, 25–34. doi: 10.1016/S0003-9861(02)00017-6 12061798

[B38] Sánchez-García A. Moreno-Peréz A. J. Muro-Pastor A. M. Salas J. J. Garces R. Martinez-Force E. (2010). Acyl-ACP thioesterase from castor (*Ricinus communis* L.): an enzymatic system appropriate for high rates of oil synthesis and accumulation. Phytochemistry 71, 860–869. doi: 10.1016/j.phytochem.2010.03.015 20382402

[B39] Schmittgen T. D. Livak K. J. (2008). Analyzing real-time PCR data by the comparative CT method. Nat. Protoc. 3, 1101–1108. doi: 10.1038/nprot.2008.73 18546601

[B40] Schnurr J. A. Shockey J. M. de Boer G. J. Browse J. A. (2002). Fatty acid export from the chloroplast. Molecular characterization of a major plastidial acyl-coenzyme A synthetase from Arabidopsis. Plant Physiol. 129, 1700–1709. doi: 10.1104/pp.003251 12177483 PMC166758

[B41] Serrano-Vega M. J. Garcés R. Martínez-Force E. (2005). Cloning, characterization and structural model of a FatA-type thioesterase from sunflower seeds (*Helianthus annuus* L.). Planta 221, 868–880. doi: 10.1007/s00425-005-1502-z 15841386

[B42] Shao Q. Liu X. F. Su T. Ma C. L. Wang P. P. (2019). New insights into the role of seed oil body proteins in metabolism and plant development. Front. Plant Sci. 10. doi: 10.3389/fpls.2019.01568 31921234 PMC6914826

[B43] Shi W. Zhang D. Ma Z. (2022). Transcriptome analysis of genes involved in fatty acid and lipid biosynthesis in developing walnut (*Juglans regia* L.) seed kernels from Qinghai Plateau. Plants 11, 3207. doi: 10.3390/plants11233207 36501246 PMC9737478

[B44] State Food and Drug Administration (2016). National food safety standard: determination of fats in foods (Beijing, China: China Standards Press), 6.

[B45] Troncoso-Ponce M. A. Kilaru A. Cao X. Durrett T. P. Fan J. Jensen J. K. . (2011). Comparative deep transcriptional profiling of four developing oilseeds. Plant J. 68, 1014–1027. doi: 10.1111/j.1365-313X.2011.04763.x 21851431 PMC3507003

[B46] Wang X. Long Y. Yin Y. Zhang C. Gan L. Liu L. . (2015). New insights into the genetic networks affecting seed fatty acid concentrations in. Brassica napus. Plant Biol. 15, 91. doi: 10.1186/s12870-015-0475-8 25888376 PMC4377205

[B47] Wang J. Ye Q. Yu N. Huan W. Sun J. Nie X. . (2021). Preparation of multiresponsive hydrophilic molecularly imprinted microspheres for rapid separation of Gardenia yellow and geniposide from Gardenia fruit. Food Chem. 374, 131610. doi: 10.1016/j.foodchem.2021.131610 34823938

[B48] Wells R. Trick M. Soumpourou E. Clissold L. Morgan C. Werner P. . (2014). The control of seed oil polyunsaturate content in the polyploid crop species *Brassica napus*. Mol. Breed. 33, 349–362. doi: 10.1007/s11032-013-9954-5 24489479 PMC3901927

[B49] Xiao W. Li S. Wang S. Ho C. T. (2017). Chemistry and bioactivity of *Gardenia jasminoides*. J. Food Drug Anal. 25, 43–61. doi: 10.1016/j.jfda.2016.11.005 28911543 PMC9333430

[B50] Yin F. Liu J. (2018). Research and application progress of *Gardenia jasminoides*. Chin. Herb. Med. 10, 362–370. doi: 10.1016/j.chmed.2018.09.001 38826717

[B51] Zhang N. Bian Y. Yao L. (2022). Essential Oils of *Gardenia jasminoides* J. Ellis and *Gardenia jasminoides* F. Longicarpa Z.W. Xie & M. Okada flowers: chemical characterization and assessment of anti-inflammatory effects in alveolar macrophage. Pharmaceutics 14, 966. doi: 10.3390/pharmaceutics14050966 35631552 PMC9145545

[B52] Zhang M. Fan J. Taylor D. C. Ohlrogge J. B. (2009). DGAT1 and PDAT1 acyltransferases have overlapping functions in Arabidopsis triacylglycerol biosynthesis and are essential for normal pollen and seed development. Plant Cell. 21, 3885–3901. doi: 10.1105/tpc.109.071795 20040537 PMC2814504

[B53] Zhang Z. Tong Z. Shao Y. Su G. Li K. Li C. (2024). Comparing and evaluating the oil composition of olive oil of 85 olive varieties in the liangshan region, China. Agronomy 14, 304. doi: 10.3390/agronomy14020304 30654563

[B54] Zhao Y. Wang Y. Huang Y. Cui Y. Hua J. (2018). Gene network of oil accumulation reveals expression profiles in developing embryos and fatty acid composition in upland cotton. J. Plant Physiol. 228, 101–112. doi: 10.1016/j.jplph.2018.06.002 29886195

[B55] Zhou X. Jiang L. Li P. Chen J. Chen Y. Yang Y. . (2024). The biosynthesis pattern and transcriptome analysis of *Sapindus saponaria* oil. Plants 13, 1781. doi: 10.3390/plants13131781 38999621 PMC11244568

[B56] Zhou L. Wu Q. Yang Y. Li Q. Li R. Ye J. (2024). Regulation of oil biosynthesis and genetic improvement in plants: Advances and prospects. Genes 15, 1125. doi: 10.3390/genes15091125 39336716 PMC11431182

[B57] Zhu Z. Zhang S. Liu H. Shen H. Lin X. Yang F. . (2012). A multi-omic map of the lipid-producing yeast Rhodosporidium toruloides. Nat. Commun. 3, 1112. doi: 10.1038/ncomms2112 23047670 PMC3493640

