# Correction: Transcriptome-based analysis of oil accumulation pattern and key gene screening in *Gardenia jasminoides* fruits

**DOI:** 10.3389/fpls.2026.1885258

**Published:** 2026-06-10

**Authors:** Liu Su, Jiwu Cao, Yunzhu Chen, Peiwang Li, Jingzhen Chen, Changzhu Li, Qiang Liu, Zhihong Xiao, Huifang Cao, Ding Kuang, Aihong Wu, Yudong Lu, Xiao Zhou, Yan Yang

**Affiliations:** 1 State Key Laboratory of Utilization of Woody Oil Resource, Hunan Academy of Forestry, Changsha, China; 2 College of Forestry, Central South University of Forestry and Technology, Changsha, China; 3 Key Laboratory of National Forestry and Grassland Administration on Utilization Science for Southern Woody Oilseed, Hunan Academy of Forestry, Changsha, China; 4 College of Environmental Engineering, Changsha Environmental Protection Vocational College, Changsha, China; 5 Hunan Haitai Bonong Biotechnology Co., Ltd., Yueyang, China; 6 Real Estate Registration Center, Xinning County Natural Resources Bureau, Xingning, China; 7 College of Chemistry and Materials Science, Fujian Normal University, Fujian, China

**Keywords:** fatty acid, *Gardenia jasminoides*, key enzyme genes, oil biosynthesis, transcriptome analysis, triacylglycerol

The **Funding** statement was incorrect in the original article. Two incorrect funders were provided: “Key Scientific and Technological Innovation Platform of Hunan Province (Grant No. 2024PT0001)” and “Innovation Demonstration Project of Chenzhou City (Grant No. 2022sfq53).” The funders “Special Project of the State Key Laboratory of Woody Oil Resources Utilization, Ministry of Science and Technology” (which has no project number) and “State Key Laboratory of Woody Oil Resources Utilization (2025ZYT027) to author LS” were erroneously omitted.

The original statement was as follows:

“The author(s) declared that financial support was received for this work and/or its publication. This work was financially supported by the Key Scientific and Technological Innovation Platform of Hunan Province (Grant No. 2024PT0001), the Natural Science Foundation of Hunan Province (Grant Nos. kq2208098 and kq2208099), the Innovation Demonstration Project of Chenzhou City (Grant No. 2022sfq53), the Science and Technology Plan Major Project of Changsha City (Grant No. kq2301004), the Forestry Technology Research and Innovation Projects (Grant Nos. XLK202410 and XLK202411), the National Key Research and Development Project (Grant No. 2024YFB4205901), the Central Forestry Promotion Demonstration Project (Grant Nos. [2023]XT06 and [2023]XT28), the Yuelushan Laboratory Talent Program (Grant No. 2025RC2087), and the National Improved Variety Demonstration Base Projects for Cornus wilsoniana and Litsea cubeba.”

The correct **Funding** statement is as follows:

“The author(s) declared that financial support was received for this work and/or its publication. This work was financially supported by the Special Project of the State Key Laboratory of Woody Oil Resources Utilization, Ministry of Science and Technology, the State Key Laboratory of Woody Oil Resources Utilization (Grant No. 2025ZYT027), the Natural Science Foundation of Hunan Province (Grant Nos. kq2208098 and kq2208099), the Science and Technology Plan Major Project of Changsha City (Grant No. kq2301004), the Forestry Technology Research and Innovation Projects (Grant Nos. XLK202410 and XLK202411), the National Key Research and Development Project (Grant No. 2024YFB4205901), the Central Forestry Promotion Demonstration Project (Grant Nos. [2023]XT06 and [2023]XT28), the Yuelushan Laboratory Talent Program (Grant No. 2025RC2087), and the National Improved Variety Demonstration Base Projects for *Cornus wilsoniana* and *Litsea cubeba*.”

