# Supplementary figures and images for: Transcriptome-based analysis of oil accumulation pattern and key gene screening in Gardenia jasminoides fruits
Source: Front Plant Sci. 2026 Mar 6;17:1774066. doi: 10.3389/fpls.2026.1774066 (PMC13002815; doi:10.3389/fpls.2026.1774066)

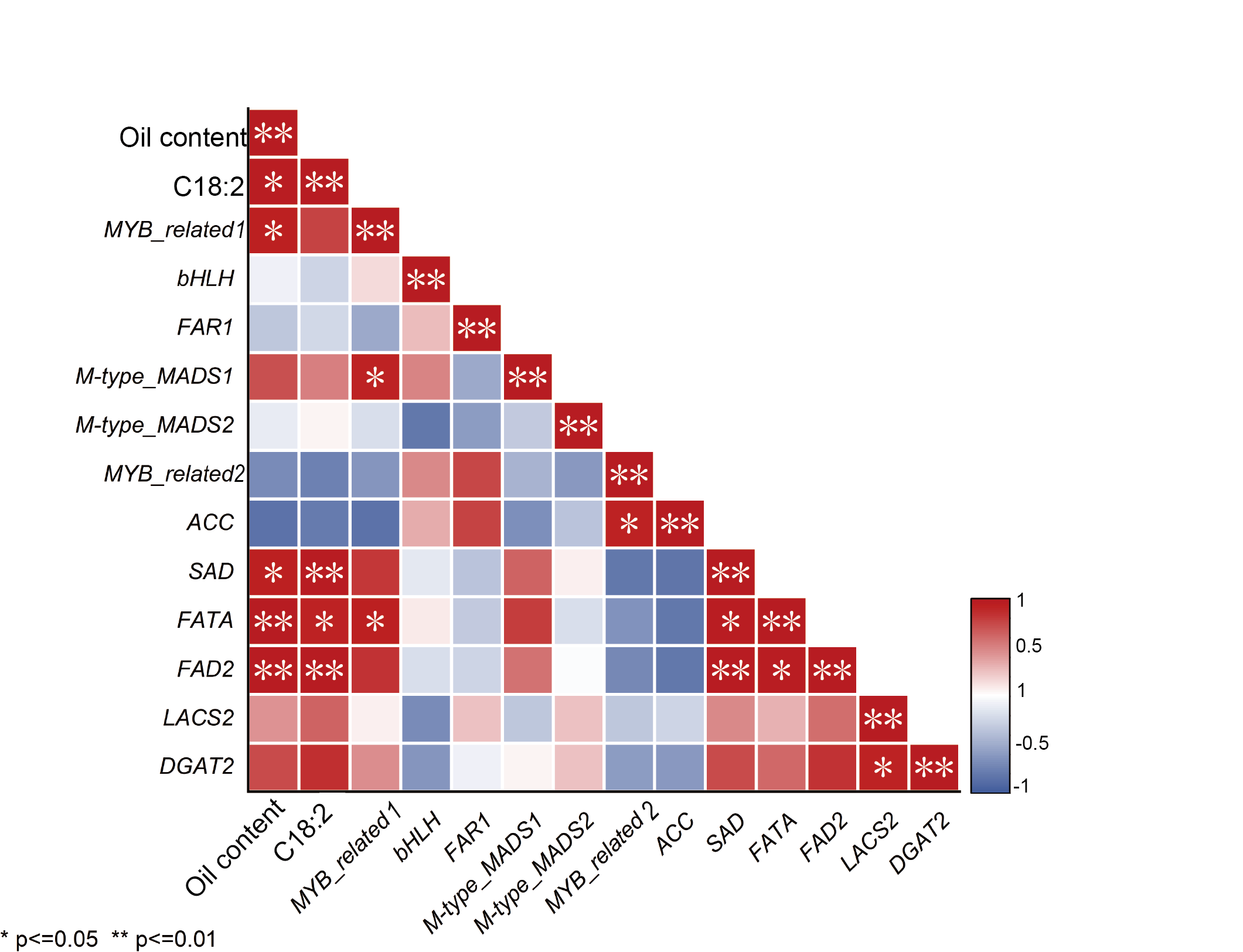

Supplement: Supplementary file 1 [file Image1.tif]
